# Impact of early postoperative activities on postoperative recovery in patients undergoing abdominal surgery: A protocol for systematic review and meta-analysis

**DOI:** 10.1097/MD.0000000000027556

**Published:** 2021-11-05

**Authors:** Aohua Fang, Wei Ding, Wei Zeng, Jinman Zhou, Hongfang Zhu, Jiaohua Yan, Na Wang

**Affiliations:** Department of Nursing, Wuhan Wuchang Hospital, Wuchang Hospital Affiliated to Wuhan University of Science and Technology, Hubei Province Key Laboratory of Occupational Hazard Identification and Control, Wuhan University of Science and Technology, Wuhan, Hubei, China.

**Keywords:** abdominal surgery, early postoperative activities, meta-analysis, postoperative recovery, postsurgical actions, protocol

## Abstract

**Introduction:**

A refined nursing process is utilized to formulate a detailed early postsurgical activity plan. The postsurgical activity aims to conduct focused and planned interventions to address the early postoperative activities of patients, enhance the awareness and compliance of the patients through the early postsurgical activities. Currently, in traditional clinical practice, there is no clear evidence showing the effect of initial postsurgical actions related to the rehabilitation of inpatients undertaking abdominal operations. The present study will systematically evaluate how initial postsurgical actions impact the rehabilitation of patients undertaking abdominal operation through the analysis of relevant domestic and foreign literature.

**Objective:**

Analyze the how initial postsurgical actions impact the rehabilitation of abdominal surgery inpatients.

**Methods and analysis:**

The present systematic study will retrieve randomized controlled trials and case-control studies from online databases. The retrieved studies will describe the initial postsurgical activities in inpatients undergoing abdominal surgery. Accordingly, the following databases are searched for the aforementioned types of studies: Cochrane library, China National of Knowledge Infrastructure, Web of Science, PubMed database, WanFang database, and Embase database. Studies from inception to August 19, 2021 will be searched. The quality evaluation and data extraction for the studies that will satisfy the inclusion criteria will be conducted by 2 independent researchers. A meta-analysis on the postoperative indicators will be performed using RevMan 5.3.5 software.

## 1 Introduction

Abdominal surgery is one of the most commonly performed surgeries in clinical practices. It refers to surgery on abdominal organs to restore and treat some diseases, such as digestive tract diseases, abdominal abscess, peritonitis, and accidental injuries.^[1]^ There is a high incidence rate of postsurgical complications associated with abdominal surgeries, these include abdominal distension, lung infections, venous thrombosis, and intestinal adhesions. Some believe that in the postabdominal surgical period, there is a high likelihood for the vitality to be severely damaged, and that inpatients should remain in bed in the immediate period following surgery. It is common for people to be afraid of pain from wounds or worry about accidental wound openings, falls, drainage tube prolapse, etc. As a result, most prefer to stay in bed or lie in a stationary position, which has an impact on the effect of rehabilitation.^[2,3]^ Reportedly, long-term bed rest in such instances will reduce the muscle strength of the entire body, affect the lung functionality and oxidation capacity of tissues, leading to an elevated risk of venous thrombosis.^[4]^ Early postsurgical activities can elevate gastrointestinal motility and encourage the recovery of the digestive system, which is conducive to promoting the patient's postsurgical food intake. Besides, increasing nutrient intake also promotes wound healing.^[5]^ Simultaneously, early postoperative activities have a positive impact on the mental wellbeing of the patient and helps restore the patient's self-confidence.^[6]^ Admittedly, numerous randomized controlled trials (RCTs) have reported that early postsurgical actions have a positive influence on improving the rehabilitation of patients in the postsurgery period.^[7]^ However, there are contradicting results due to variations in nursing content and research programs. Concurrently, there is no uniformity in the timing and time of early postsurgical actions. Moreover, there is an inadequacy in dependable, consistent evidence-based outcomes, which further restricts research outcomes. Thus, the present systematic analysis will assess how early postsurgical actions impact inpatients who are subject to abdominal surgeries.

## 2 Methods and analysis

### 2.1 Search strategy process

A computer will be used to perform a comprehensive search online databases to retrieve RCTs and case-control studies. The retrieved studies will describe the initial postsurgical activities in inpatients undergoing abdominal surgery. Accordingly, the following databases are searched for the aforementioned types of studies: Cochrane library, China National of Knowledge Infrastructure, Web of Science, PubMed database, WanFang database, and Embase database. Studies from inception to August 19, 2021 will be included in the search. The languages are English and Chinese. The following keywords are used in the search: early postoperative activities, early activities, early out-of-bed activities, and abdominal surgery.

### 2.2 Statistical analysis

The present study will employ Rev Man 5.3 software to conduct meta-analysis on the data. Accordingly, the odds ratio and 95% confidence interval will be calculated for binary variable indicators. Meanwhile, the mean difference and 95% confidence interval will be calculated for continuous variables. Moreover, this study will employ a *χ*^*2*^ test to test the heterogeneity of the included studies. In the case where *P* ≥ .1 and *I*^*2*^ < 50%, the authors will consider the considered studies to be less heterogeneous, in which case a fixed model will be adopted for analysis. Alternatively, if *P* < .1, and *I*^*2*^ ≥ 50%, the authors will consider substantially high heterogeneity, in which case a random model is more suitable to facilitate the analysis. The test standard (α) is 0.05, and when *P* < .05, the difference is considered to be statistically significant.

### 2.3 Eligibility criteria

#### 2.3.1 Inclusion criteria

(1) The research object involve patients undergoing abdominal surgery.
(2) The study is either a RCT or a case-controlled trial.
(3) The experimental group adopts early postoperative activities, whilst the control group adopts traditional postoperative activities.
(4) The study describes the early postoperative activities related to both the experimental group and control group.
(5) Observation of the outcome must include 1 or more of the following postoperative recovery indicators: first postoperative exhaust time, first postoperative defecation time, postoperative pain score, postoperative sleep time, incidence of gastrointestinal discomfort.

#### 2.3.2 Exclusion criteria

(1) Uncontrolled studies such as reviews, case reports, and single cohorts.
(2) Repeated publications for the same research population.
(3) Incomplete clinical data of the cases.

### 2.4 Data extraction

Two researchers will read the full text of the included studies, and independently extract data according to the pre-established plan. In the case of any disputes/disagreements, the authors will consult a third independent researcher for further evaluation. The primary extraction content includes the general information of the included research (title of study, first author, publication year, and source of study); research methods (research plan design, sample size, characteristics of the research population, and nursing process), and observation outcomes (first exhaust time after operation, first defecation time after the surgery, postoperative pain score, postoperative sleep time, and gastrointestinal discomfort).

### 2.5 Quality appraisal

Two investigators will independently evaluate all the included studies. If there is a disagreement, a third researcher will participate in resolving the disagreement. Two investigators will evaluate the included RCTs based on the Cochrane Risk of Bias Evaluation Tool.

## 3 Discussion

In the immediate period following abdominal surgery, many patients are reluctant to move around, most fear discomfort, pains from wounds, or some organ dysfunctions. Sometimes, it can persist for several days after the operation, some would not even dare to turn over, let alone exercise in or out of bed. However, in reality, it will easily lead to some postoperative complications, which will increase recovery time of patients after surgery. For example, 2 major comorbidities of the respiratory system are falling pneumonia and carbon dioxide retention, both are the result of long-term bed rest. Besides, orthostatic hypotension and thrombosis are common cardiovascular effects of long-term bedridden patients. Unless completely immobilized, patients can improve circulation speed to the legs by moving early after an operation, particularly after abdominal surgery. Most importantly, it can avoid or reduce the incidence of thrombosis. Due to reduced activity and the consumption of diseases, patients often suffer adverse impacts on the digestive system from anorexia, particularly when the intake of fiber and water is reduced. Moreover, the peristalsis of the gastrointestinal tract slows down, leading to constipation. Other complications include urinary tract stones, urinary tract infections, and the emergence of bedsores. Admittedly, previous studies have reported that rest and sleep are necessary to recover after a surgery or an injury, especially helping to restore the physical strength consumed during surgery, reduce consumption, as well as promote protein synthesis and tissue repair. However, excessive rest and sleep can lead to aforementioned complications.^[8–10]^ Therefore, if the patient's condition permits the patient after the operation, he should follow the instructions of the doctor and nurse for early postoperative activities. However, there is no uniformity associated with the time and operation of early postoperative activities. Besides, there is an inadequacy of reliable, consistent basis based on evidence, which restricts the promotion of the plan. Therefore, the present research plan aims to systematically evaluate the effects of early postsurgery actions to guide patients towards faster recovery after undergoing abdominal surgery and obtain a reliable, standardized conclusion.

## Author contributions

**Conceptualization:** Aohua Fang, Wei Ding, Wei Zeng, Jinman Zhou, Hongfang Zhu, Jiaohua Yan.

**Data curation:** Aohua Fang, Wei Ding, Wei Zeng, Hongfang Zhu, Jiaohua Yan.

**Formal analysis:** Wei Ding, Jinman Zhou, Hongfang Zhu, Jiaohua Yan.

**Funding acquisition:** Aohua Fang, Wei Ding, Wei Zeng, Jinman Zhou, Jiaohua Yan, Na Wang.

**Investigation:** Aohua Fang, Jinman Zhou, Hongfang Zhu.

**Methodology:** Wei Zeng, Jiaohua Yan.

**Project administration:** Na Wang.

**Resources:** Aohua Fang, Wei Zeng, Jinman Zhou, Hongfang Zhu, Jiaohua Yan, Na Wang.

**Supervision:** Aohua Fang, Wei Ding, Jinman Zhou, Hongfang Zhu.

**Validation:** Aohua Fang, Wei Zeng, Jiaohua Yan, Na Wang.

**Visualization:** Wei Zeng, Jiaohua Yan.

**Writing – original draft:** Aohua Fang, Wei Zeng, Jiaohua Yan.

**Writing – review & editing:** Jiaohua Yan, Na Wang.
